# Peroxo-Containing Heteropolyanions [{XW_3_O_7_(O_2_)_2_}_2_O]^6–^ (X = HPO_4_^2–^, HAsO_4_^2–^, CH_3_AsO_3_^2–^): Synthesis, Structure, and Antibacterial Properties

**DOI:** 10.1021/acs.inorgchem.6c01165

**Published:** 2026-05-19

**Authors:** Sahar Khandan, Anupam Sarkar, Arun Pal, Friedrich Matteo Lüderitz, Marziyeh Kianihaftlang, Ayush Kant Ranga, Levente Kiss, Matthias S. Ullrich, Arnulf Materny, Cristian Silvestru, Ulrich Kortz

**Affiliations:** † School of Science, 84498 Constructor University, Campus Ring 1, Bremen 28759, Germany; ‡ Department of Chemistry, Supramolecular Organic and Organometallic Chemistry Centre (SOOMCC), Faculty of Chemistry and Chemical Engineering, 54741 Babeş-Bolyai University, 11 Arany Janos, Cluj-Napoca 400028, Romania

## Abstract

We report the synthesis of the peroxo-containing heteropolyanions [{XW_3_O_7_(O_2_)_2_}_2_O]^6–^ (X = HPO_4_^2–^, HAsO_4_^2–^, CH_3_AsO_3_^2–^) obtained in aqueous medium via a straightforward one-pot reaction in the presence of H_2_O_2_. Single-crystal X-ray diffraction analysis reveals that all polyanions adopt dimeric structures composed of two {XW_3_} subunits interconnected by three oxo bridges. In each {XW_3_} unit, two peroxo groups are coordinated to tungsten centers situated in adjacent edge-sharing pentagonal bipyramids, whereas the third tungsten center adopts a regular octahedral environment bearing exclusively terminal oxo ligands. The solution stability of the polyanions was systematically investigated over a range of pH values and time intervals by multinuclear NMR spectroscopy. The antibacterial screening of the peroxo-tungstoarsenate­(V) and its organoarsonate­(V) derivative was performed against representative Gram-negative and Gram-positive bacterial strains.

## Graphical abstract

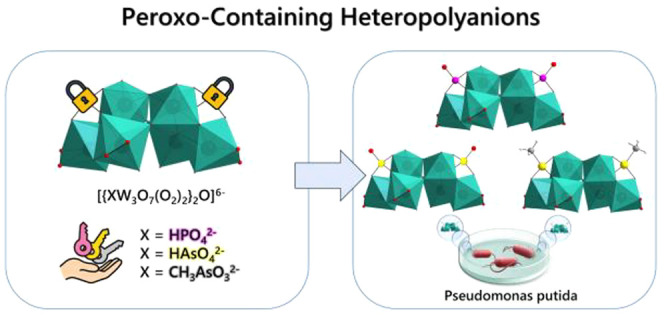

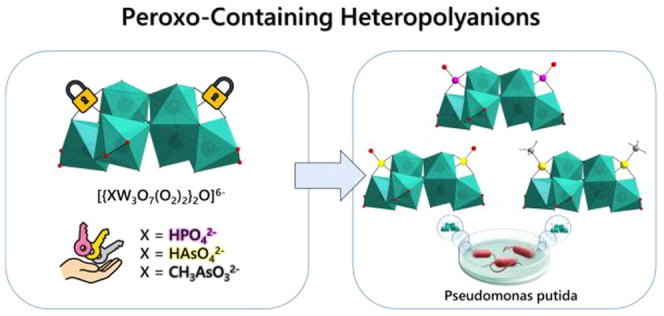

## Introduction

Polyoxometalates (POMs) are a structurally versatile class of anionic metal-oxo clusters, composed of high-valent early transition metals, most commonly W^VI^, Mo^VI^, and V^V^. Their assemblies are usually based on oxo-bridging, mainly corner- and edge-shared octahedra, forming vast families of discrete anions.^1^ Beyond their structural variety, POMs are tunable entities with a remarkable capacity for use in catalytic, electronic, and biomedical systems.^2^ One of the most significant developments in POM chemistry was the incorporation of peroxo groups, leading to the emergence of peroxo-POMs as a distinct subclass. These species are typically obtained by reacting preformed (parent or lacunary) polyoxoanions or simple metal precursors with H_2_O_2_.^3,4^ Depending on the precursor and reaction conditions, both isopoly and heteropolyanion frameworks can accommodate one or more peroxo groups. The first isolated peroxo-containing isopolytungstate, reported by Einstein and Penfold in 1964, is the dinuclear complex [W_2_O_3_(O_2_)_4_(H_2_O)_2_]^2–^.^4a^ Other examples include the mononuclear [W­(O_2_)_4_]^2–^,^4b^ as well as tetranuclear species such as [W_4_O_8_(O_2_)_6_(CO_3_)]^6–^,^4c^ [W_4_O_6_(O_2_)_6_(OH_2_)­(H_2_O)_2_]^2–^,^4d^ [W_4_O_12_(O_2_)_2_]^4–^,^4e^ and the heptanuclear derivative [W_7_O_22_(O_2_)_2_]^6–^.^4f^

A milestone in the field of peroxo-heteropolytungstates was achieved in 1985, when Venturello and co-workers reported the synthesis and characterization of the tetrakis­(diperoxotungsto)­phosphate anion [PO_4_{WO­(O_2_)_2_}_4_]^3–^ (**PW**_**4**_).^5^ The structure features a central tetrahedral PO_4_^3–^ heterogroup surrounded by four {WO­(O_2_)_2_} fragments. Each tungsten center is coordinated by two peroxo ligands in a cis-η^2^ fashion. The crystal structure of the arsenate analogue [AsO_4_{WO­(O_2_)_2_}_4_]^3–^ (**AsW**_**4**_) was later reported by Brégeault’s team.^3d^ These so-called Venturello-type species not only expanded the structural scope of POMs but also exhibited remarkable activity in H_2_O_2_-based oxidation catalysis.^3a–3d,6^ Later, in 1991, Iwamoto and co-workers published the synthesis and crystal structure of the dimeric polyanion [{H­(SO_4_)­W_3_O_7_(O_2_)_2_}_2_O]^4–^ (**S**_**2**_ **W**_**6**_), revealing two nearly identical diperoxotritungstosulfate units bridged by three μ_3_-oxo ligands.^7^ In 1995, Griffith and co-workers isolated an organophosphonate-functionalized peroxotungstate [(CH_3_PO_3_)­{CH_3_PO_2_(OH)}­W_6_O_13_(O_2_)_4_(OH)_2_(OH_2_)]^3–^,^8^ structurally related to Iwamoto’s compound. More recently, other comparable species such as [P_3_W_6_(O_2_)_6_(OH)_2_O_22_]^7–^,^9a^ [M_2_(H_2_O)_4_(OH)­P_2_W_6_(O_2_)_4_O_23_]^3–^ (M = Pr, Sm) and [M­(H_2_O)_4_P_4_W_6_(O_2_)_6_(OH)_4_O_24_]^5–^ (M = Gd, Tb, Dy, Ho) have been reported by Zhang’s group.^9b^

Since 2008, our research group has systematically investigated the synthesis and characterization of diverse peroxo-POMs.^10^ Building on this ongoing interest, herein, we report on the synthesis of three novel peroxo-containing heteropolyanions and their antibacterial activity.

## Experimental Section

### General Methods and Materials

All reagents were used as received without further purification. Orthoarsenic acid (H_3_AsO_4_) was prepared according to a published procedure,^11^ and its molarity was determined by acid–base titration (see Supporting Information for details). Fourier transform infrared (FT-IR) spectra of solid samples were recorded on a SHIMADZU IRSpirit spectrometer in the range 4000–400 cm^–1^ using KBr pellets (32 scans, 4 cm^–1^ resolution). Peak intensities are abbreviated as w (weak), m (medium), s (strong), and sh (shoulder). Thermogravimetric analysis (TGA) was performed on a TA Instruments SDT Q600 thermobalance under nitrogen flow, with the temperature ramped from room temperature to 500 °C at 5 °C/min (Figure S1). Elemental analyses were carried out at Zentrallabor, Technische Universität Hamburg (Na, K, P, As, W) and at Analytische Laboratorien, Industriepark Kaiserau, Lindlar, Germany (C, H). Nuclear magnetic resonance (NMR) spectra were measured on a JEOL ECS 400 MHz spectrometer. ^1^H, ^13^C­{^1^H}, and ^31^P­{^1^H} NMR spectra were recorded with a 5 mm probe at resonance frequencies of 400.5 MHz (^1^H), 100.7 MHz (^13^C), and 161.8 MHz (^31^P). Chemical shifts were referenced to tetramethylsilane (^1^H, ^13^C) and 85% H_3_PO_4_ (^31^P). ^183^W NMR spectra were acquired at room temperature on the same instrument using a 10 mm probe at 16.6 MHz, with chemical shifts referenced to 1 M Na_2_WO_4_ in H_2_O.

### Synthesis of K_5_Na­[{(HPO_4_)­W_3_O_7_(O_2_)_2_}_2_O]·41H_2_O (KNa-P_2_W_6_)

Na_2_WO_4_·2H_2_O (1.00 g, 3.00 mmol) was dissolved in 20 mL of 1 M KCl and heated to 50 °C. H_3_PO_4_ (1 mL, 1.00 mmol, 1 M) was added dropwise to the tungstate solution, and the pH was adjusted to 6 with 12 M HCl. Subsequently, H_2_O_2_ (1 mL, 30%) was added, affording a light-yellow solution that was stirred at 50 °C for 1 h. Then, the pH of the solution was readjusted to 6 with 4 M KOH. In cases where a small amount of white precipitate appeared, it was removed by filtration, and the clear filtrate was allowed to stand undisturbed for crystallization. Slow evaporation of the reaction solution at room temperature yielded colorless rod-shaped crystals of **KNa-P**_**2**_ **W**_**6**_ after 2–4 days, which were collected by filtration, washed with a small amount of cold water (2 mL), and air-dried (Figure S2a). Isolated yield: 0.44 g (34%). Elemental analysis (%): calcd. Na 0.87, P 2.36, K 7.44, W 42.13; found Na 1.03, P 2.35, K 7.30, W 42.20. FT-IR (1% KBr disk/cm^–1^): 3481 (s), 1640 (w), 1109 (m), 1026 (w), 998 (w), 974 (sh), 935 (s), 921 (sh), 866 (m), 822 (w), 764 (w), 706 (w), 587 (m), 540 (m), 444 (w).

### Synthesis of K_6_[{(HAsO_4_)­W_3_O_7_(O_2_)_2_}_2_O]·29H_2_O (K-As_2_W_6_)

The synthesis of this compound was identical to that of **KNa-P**_**2**_ **W**_**6**_, but H_3_AsO_4_ (0.43 mL, 1.00 mmol, 2.3 M) was used instead of H_3_PO_4_. The colorless rod-shaped crystals of **K-As**_**2**_ **W**_**6**_ formed within 2–4 days, which were collected by filtration, washed with a small amount of cold water (2 mL), and air-dried (Figure S2b). Isolated yield: 0.60 g (48%). Elemental analysis (%): calcd. As 5.97, K 9.32, W 44.01; found As 5.66, K 9.28, W 44.10. FT-IR (1% KBr disk/cm^–1^): 3440 (s), 1637 (w), 965 (sh), 953 (w), 864 (s), 825 (s), 740 (w), 699 (w), 624 (sh), 590 (sh), 550 (m), 533 (sh), 487 (m), 439 (sh).

### Synthesis of K_3.3_Na_2.7_[{(CH_3_AsO_3_)­W_3_O_7_(O_2_)_2_}_2_O]·16H_2_O (KNa-Me_2_As_2_W_6_)

The synthesis of this compound was identical to that of **KNa-P**_**2**_ **W**_**6**_, but Na_2_CH_3_AsO_3_ (0.18 g, 1.00 mmol) was used instead of H_3_PO_4_. Colorless block-shaped crystals of **KNa-Me**_**2**_ **As**_**2**_ **W**_**6**_, suitable for single-crystal XRD analysis, were obtained after 2 weeks and isolated by filtration followed by washing with cold water (2 mL) and air-drying (Figure S2c). Notably, bulk crystallization occurred within a shorter period (approximately 4 days) when the solvent volume was reduced to 10 mL. Isolated yield: 0.66 g (56%). Elemental analysis (%): calcd. H 1.72, C 1.07, Na 2.78, K 5.77, As 6.73, W 49.57; found H 1.33, C 1.16, Na 2.75, K 5.22, As 6.20, W 49.50. FT-IR (1% KBr disk/cm^–1^): 3453 (s), 1637 (m), 1404 (w), 1290 (sh), 970 (m), 945 (sh), 884 (m), 859 (s), 828 (s), 792 (s), 750 (sh), 695 (w), 644 (w), 612 (w), 593 (sh), 537 (m), 494 (sh), 422 (sh).

### Single-Crystal X-ray Diffraction

Data acquisition was performed on a Rigaku XtaLAB Synergy single-crystal diffractometer, configured with Dualflex and HyPix detection systems, and employing kappa geometry. The instrument utilized a graphite monochromator with a MoKα radiation source (λ = 0.71073 Å). The collection of crystallographic data was facilitated by the CrysAlisPro software package.^12^ Crystals were mounted on Hampton cryoloops using Paratone-N oil and measured at 100 K. An empirical absorption correction was applied using the ABSPACK program to account for absorption effects.^13^ An initial structure solution was achieved using the Olex2 program and refined on F^2^ using the SHELX program package, employing a full-matrix least-squares approach. The SQUEEZE method in PLATON was employed for the structures with disordered solvent molecules.^14^ Crystal structure illustrations were generated using Diamond, version 3.2 (Crystal Impact GbR). Crystallographic data for all compounds are summarized in Table 1. Further details on the crystal structure investigations can be obtained free of charge under CCDC 2534480 (for **KNa-P**_**2**_ **W**_**6**_), 2534481 (for **K-As**_**2**_ **W**_**6**_) and 2534482 (for **KNa-Me**_**2**_ **As**_**2**_ **W**_**6**_) from The Cambridge Crystallographic Data Center via http://www.ccdc.cam.ac.uk/data_request/cif.

**Table 1:** Crystallographic Data for KNa-P_2_W_6_, K-As_2_W_6_, and KNa-Me_2_As_2_W_6_

	**KNa-P_2_W_6_**	**K-As_2_W_6_**	**KNa-Me_2_As_2_W_6_**
formula^a^	K_5_Na[{(HPO_4_)W_3_O_7_(O_2_)_2_}_2_O]·41H_2_O	K_6_[{(HAsO_4_)W_3_O_7_(O_2_)_2_}_2_O]·29H_2_O	K_3.3_Na_2.7_[{(CH_3_AsO_3_)W_3_O_7_(O_2_)_2_}_2_O]·16H_2_O
formula weight^a^ (g/mol)	2623.62	2507.49	2226.27
crystal system	monoclinic	monoclinic	monoclinic
space group	*P*2_1_/*n*	*C*2/*c*	*P*2_1_ */n*
*a* (Å)	29.0044 (5)	28.8924 (4)	12.21203 (13)
*b* (Å)	10.1425 (1)	10.2220 (1)	19.25368 (19)
*c* (Å)	30.1852 (5)	28.9894 (4)	16.67469 (17)
β (°)	112.206 (2)	96.646 (1)	100.383 (1)
volume (Å^3^)	8221.2 (2)	8504.14 (19)	3856.46 (7)
*Z*	1	2	4
D _calc_ (g/cm^3^)	3.377	3.342	3.678
absorption coefficient (mm^–1^)	17.53	18.41	20.01
crystal size (mm)	0.14 × 0.12 × 0.10	0.15 × 0.13 × 0.11	0.13 × 0.09 × 0.07
F (000)	7462.1	7600	3805
reflections used [*I* > 2σ (*I*)]	12396	5878	5901
independent reflections	13676	7075	6413
*R*_int_	0.089	0.083	0.102
goodness-of-fit on F^2^	1.12	1.05	1.05
*R*_1_ [I > 2σ (I)]^b^	0.055	0.032	0.035
w*R*_2_ ^c^ (all data)^c^	0.111	0.081	0.091

a The entries are the actual formula units and weights as obtained from elemental analysis on bulk samples.

b *R*_1_ = Σ||*F*_o_| – |*F*_c_||/Σ|*F*_o_|.

c *wR*_2_ = [Σ*w* (*F*_o_^2^ – *F*_c_^2^)^2^/Σ*w* (*F*_o_^2^)^2^]^1/2^.

### Raman Spectroscopy

Solid-state Raman spectra were acquired using a B&W Tek i-Raman Plus compact spectrometer equipped with a fiber-optic probe for both laser excitation and signal collection, along with a charge-coupled device (CCD) detector covering the spectral range 65–3350 cm^–1^. The instrument incorporates an internal 785 nm near-infrared diode laser and a fixed low-groove-density grating, allowing spectra to be collected across a wide range (up to 3350 cm^–1^) within a single acquisition window. The optical fiber delivering the laser beam can also be coupled to a collimator tube, enabling integration with a microscope for detailed sample inspection and Raman analysis through a microscope objective. A 50× objective was used, resulting in a laser spot size of approximately 30–35 μm. The spectral resolution of the system was about 4 cm^–1^. Wavenumber calibration was confirmed using the toluene ring-breathing mode at 1003.7 cm^–1^. Raman measurements were performed (100 mW power, 4000 ms exposure time) over the range 400–1200 cm^–1^, and each spectrum was recorded three times per spectral window.

### Antibacterial Activity: Determination of Minimal Inhibitory Concentrations (MICs) for Bacterial Cells

The antibacterial activity assays were conducted using standard microdilution techniques. A single bacterial colony was inoculated into 20 mL of the Luria–Bertani (LB) medium and incubated overnight at 37 °C. The resulting culture was adjusted to an optical density (OD_600_) corresponding to 2 × 10^6^ CFU/mL. Stock solutions of **K-As**_**2**_ **W**_**6**_ and **KNa-Me**_**2**_ **As**_**2**_ **W**_**6**_ were prepared in sterile deionized water and subsequently diluted to a working concentration of 1000 μg/mL. The minimum inhibitory concentration (MIC) was determined using 2-fold serial dilutions prepared in 96-well microtiter plates, with each sample tested in triplicate to ensure reproducibility. Following the addition of bacterial suspensions and POM solutions, plates were incubated for 24 h at 28 °C for *Bacillus* strains, and at 37 °C for all other bacterial species. Bacterial growth was evaluated by visual inspection of turbidity, followed by quantitative assessment using resazurin as a fluorometric indicator.

## Results and Discussion

### Synthesis and Structure

The peroxo-containing 6-tungsto-2-phosphate­(V) [{(HPO_4_)­W_3_O_7_(O_2_)_2_}_2_O]^6–^ (**P**_**2**_ **W**_**6**_) was synthesized in aqueous solution (pH 6) in the presence of H_2_O_2_, under stirring at 50 °C for 1 h. The polyanion was isolated as a hydrated mixed potassium–sodium salt, K_5_Na­[{(HPO_4_)­W_3_O_7_(O_2_)_2_}_2_O]·41H_2_O (**KNa-P**_**2**_ **W**_**6**_). Single-crystal X-ray analysis revealed that **KNa-P**_**2**_ **W**_**6**_ crystallizes in the monoclinic space group *P2*_*1*_ */n* and is isostructural to the earlier reported **S**_**2**_ **W**_**6**_ polyanion of Iwamoto (Figure 1).^7^ Crystallographic and refinement details are provided in Table 1.

**Figure 1:**
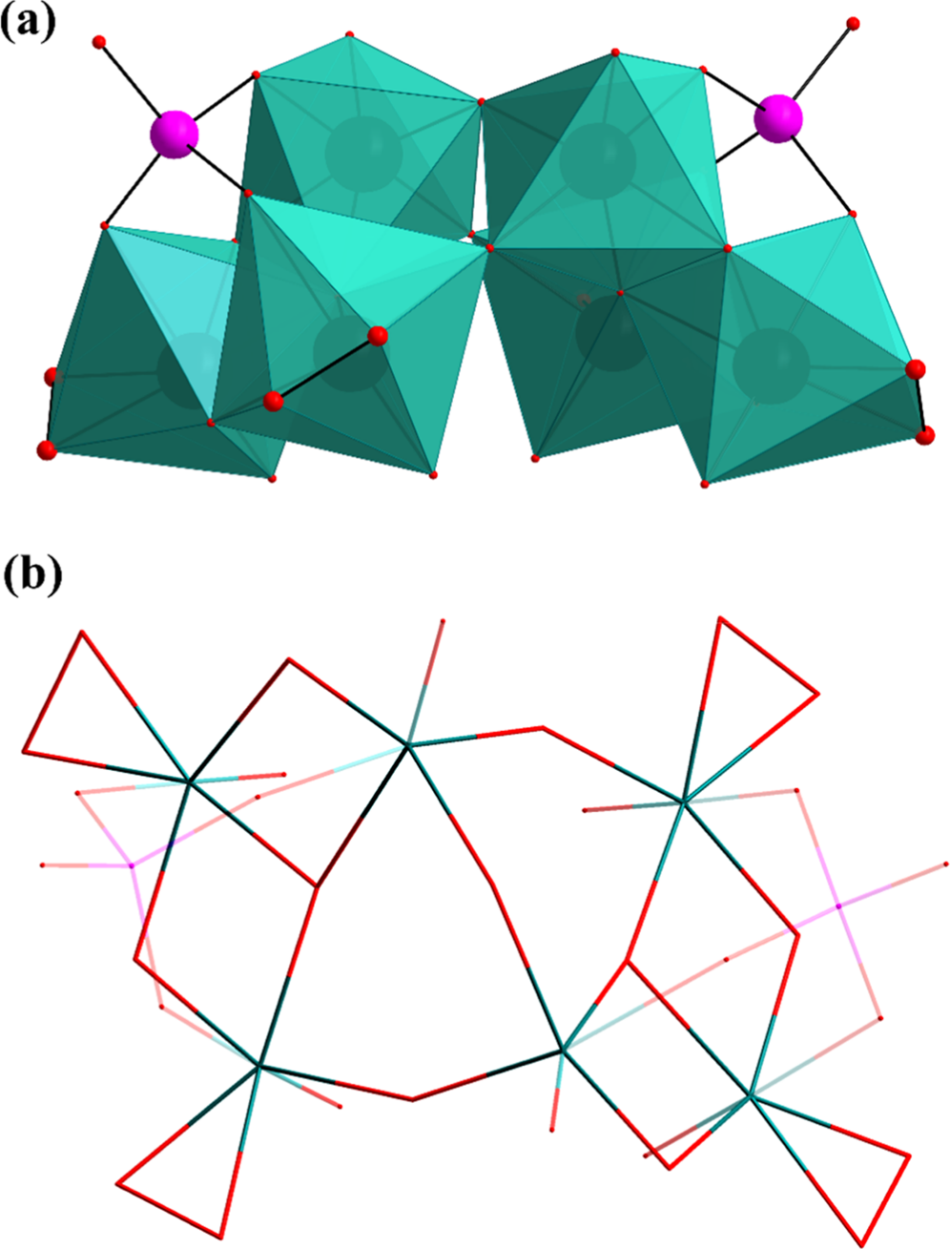
Combined ball-and-stick/polyhedral representations of the polyanions [{(HXO_4_)­W_3_O_7_(O_2_)_2_}_2_O]^6–^ (X = P, **P**_**2**_ **W**_**6;**_ As, **As**_**2**_ **W**_**6**_): (a) side view and (b) top view. Color code: P/As (pink), W (green), O (red). In (a), the peroxo oxygen atoms are depicted with enlarged radii for emphasis. In (b), selected X–O and W–O bonds of the polyanion are shown with reduced opacity to enhance structural visualization.

As shown in Figure 1, the polyanion **P**_**2**_ **W**_**6**_ adopts a dimeric structure, composed of two {PW_3_} subunits connected to each other by three μ_2_-oxo bridges. In each subunit, two peroxo groups are bonded to tungsten centers in two adjacent edge-shared {WO_7_} pentagonal bipyramids. The remaining tungsten atom is hexa-coordinated in a regular octahedral fashion and has only one terminal oxygen. The three tungsten centers are connected to each other by a μ_3_-oxo bridge. The average O–O bond length in **P**_**2**_ **W**_**6**_ is 1.48(6) Å, which is comparable to that observed in previously reported peroxotungstates.^3d,4,5,7–9^ Additionally, the W–O_peroxo_ bond lengths in **P**_**2**_ **W**_**6**_ (1.95(1)–1.97(8) Å) are slightly longer than those in **S**_**2**_ **W**_**6**_ (1.90(0)–1.94(6) Å).^7^ The sulfur centers in **S**_**2**_ **W**_**6**_, residing in a higher formal oxidation state and exhibiting a stronger electron-withdrawing character than the phosphorus centers in **P**_**2**_ **W**_**6**_, induce a more pronounced depletion of electron density at the tungsten sites. This electronic modulation strengthens the W–O_peroxo_ interactions, as reflected by the shorter W–O bond distances observed crystallographically. Both {PO_4_} heterogroups in the structure of **P**_**2**_ **W**_**6**_ have tetrahedral geometry and adopt tripodal coordination modes. The P–O bond lengths fall within the range of 1.50(9)−1.57(2) Å, and the O–P–O bond angles vary from 105.82° to 113.15°. Bond valence sum (BVS) calculations confirm the +5 oxidation state of the phosphorus centers and monoprotonation of their terminal oxygens (O4P1 and O4P2) in the **P**_**2**_ **W**_**6**_ polyanion (Table S1).^15^ The {PW_3_} subunits give polyanion **P**_**2**_ **W**_**6**_ an overall charge of −6, which is stabilized in the solid state by K^+^ and Na^+^ counter cations.

We have also synthesized the polyanion [{(HAsO_4_)­W_3_O_7_(O_2_)_2_}_2_O]^6–^ (**As**_**2**_ **W**_**6**_) in analogy to **P**_**2**_ **W**_**6**_ but using H_3_AsO_4_ under otherwise identical reaction conditions. The polyanion **As**_**2**_ **W**_**6**_ crystallizes as a hydrated potassium salt, K_6_[{(HAsO_4_)­W_3_O_7_(O_2_)_2_}_2_O]·29H_2_O (**K-As**_**2**_ **W**_**6**_), in the monoclinic space group *C*2/*c* (Figure 1, Table 1). In the structure of polyanion **As**_**2**_ **W**_**6**_, the average O–O bond length is 1.49(3) Å, typical of coordinated peroxo groups. The W–O_peroxo_ distances range from 1.95(0) to 1.97(6) Å. The arsenic atoms adopt a nearly regular tetrahedral geometry, with As–O bonds of 1.65(1)−1.71(5) Å and O–As–O angles from 106.53° to 111.55°. BVS calculations support further assignment of the +5 oxidation state to the arsenic atoms and indicate monoprotonation of O4A1 and O4A2 terminal oxygens within the **As**_**2**_ **W**_**6**_ framework (Table S2).

In addition, we succeeded in isolating the organoarsonate­(V) derivative [{(CH_3_AsO_3_)­W_3_O_7_(O_2_)_2_}_2_O]^6–^ (**Me**_**2**_ **As**_**2**_ **W**_**6**_) using Na_2_CH_3_AsO_3_ as source for the heterogroup (Figure 2). The compound **Me**_**2**_ **As**_**2**_ **W**_**6**_ crystallizes as a hydrated mixed potassium–sodium salt K_3.3_Na_2.7_[{(CH_3_AsO_3_)­W_3_O_7_(O_2_)_2_}_2_O]·16H_2_O (**K-Me**_**2**_ **As**_**2**_ **W**_**6**_) in the monoclinic space group *P2*_*1*_ */n*, indicating slightly reduced symmetry compared to **As**_**2**_ **W**_**6**_, attributed to the incorporation of methyl groups (Table 1).

**Figure 2:**
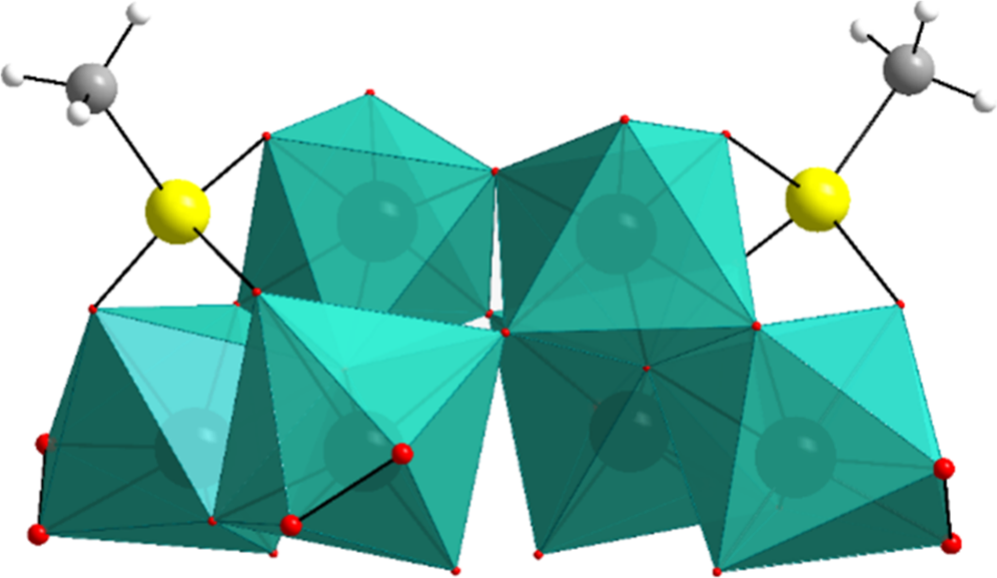
Combined ball-and-stick/polyhedral representation of [{(CH_3_AsO_3_)­W_3_O_7_(O_2_)_2_}_2_O]^6–^ (**Me**_**2**_ **As**_**2**_ **W**_**6**_). Color code: As (yellow), W (green), O (red), C (gray), and H (white). For clarity, the peroxo oxygen atoms are shown enlarged.

The **Me**_**2**_ **As**_**2**_ **W**_**6**_ ion is structural closely related to the known polyanion [(CH_3_PO_3_)­{CH_3_PO_2_(OH)}­W_6_O_13_­(O_2_)_4_­(OH)_2_­(OH_2_)]^3–^, reported by Griffith and co-workers.^8^ In the latter, the two phosphorus-containing heterogroups are chemically and crystallographically distinct: one functions as a fully deprotonated {CH_3_PO_3_} group, while the other exists as a monoprotonated {CH_3_PO_2_(OH)} unit. In contrast, the structure of **Me**_**2**_ **As**_**2**_ **W**_**6**_ features two chemically equivalent methylarsonate­(V) groups. No evidence of protonation asymmetry or mixed-valent As^III^/As^V^ species is observed, as confirmed by BVS analysis, single-crystal XRD, and NMR data (Table S3). The polyanion **Me**_**2**_ **As**_**2**_ **W**_**6**_ exhibits an average O–O bond length of 1.484 Å and W–O_peroxo_ distances of 1.95(6)–1.98(1) Å. The arsenic centers in **Me**_**2**_ **As**_**2**_ **W**_**6**_ (As–O = 1.68(2)–1.71(7) Å; O–As–O = 106.07–113.85°) are slightly more distorted than those in **As**_**2**_ **W**_**6**_ (As–O = 1.65(1)–1.71(5) Å; O–As–O = 106.53–111.55°), although both are consistent with As^V^ in tetrahedral coordination. Table S4 summarizes the average bond distances, X–O, O–O, and W–O_peroxo_, in the three polyanions.

The new peroxo-containing polyanions **P**_**2**_ **W**_**6**_, **As**_**2**_ **W**_**6**_, and **Me**_**2**_ **As**_**2**_ **W**_**6**_ were synthesized in a 1:3 molar ratio of the heteroatom source (H_3_AsO_4_, H_3_PO_4_, or Na_2_CH_3_AsO_3_) to Na_2_WO_4_. Additionally, Na_2_HAsO_4_ and Na_2_HPO_4_ also can be used as precursors for the preparation of **As**_**2**_ **W**_**6**_ and **P**_**2**_ **W**_**6**_, respectively. The mild reaction temperature of 50 °C was found to be essential for ensuring sufficient solubility of the reactants and avoiding the formation of byproducts. These are optimized conditions which allowed for the isolation of pure crystalline material, as confirmed by elemental analysis and NMR spectroscopy.

### FT-IR and Raman Spectroscopy

The FT-IR spectra were recorded on KBr pellets (1%) and the fingerprint region (1200–400 cm^–1^) is shown in Figure 3. The spectrum of **KNa-P**_**2**_ **W**_**6**_ exhibits ν­(P–O) stretching bands at 1109 and 1026 cm^–1^, characteristic of phosphate groups (Figure 3a).^16^ The bands at 998, 974, 935, and 921 cm^–1^ can be assigned to ν­(W=O) stretching vibrations, while the bands in the 866–700 cm^–1^ region correspond to bridging ν­(W–O–W) linkages involving corner- and edge-sharing polyhedral.^16a^ The spectrum of **K-As**_**2**_ **W**_**6**_ exhibits shifts of characteristic bands to lower wavenumbers, consistent with the longer and hence weaker As–O bonds relative to P–O bonds, as previously reported for phospho-versus arsenotungstates.^16b,16c^ In the spectrum of **K-As**_**2**_ **W**_**6**_ and **KNa-Me**_**2**_ **As**_**2**_ **W**_**6**_, the absorptions near 945 cm^–1^ and 953 cm^–1^ can be attributed to ν­(As–O) stretching modes, although overlap with ν­(W=O) vibrations complicates exact assignments (Figure 3b,c).^16d^ The compound **KNa-Me**_**2**_ **As**_**2**_ **W**_**6**_ shows an overall spectral pattern similar to that of **K-As**_**2**_ **W**_**6**_, with the ν­(W=O) stretching observed around 970 cm^–1^ and a comparable ν­(W–O–W) fingerprint between 860 and 420 cm^–1^. In addition, new bands in the 1400–1200 cm^–1^ region arise from δ­(C–H) bending modes of the methyl groups (see full spectra provided in Figure S3).^16e^ As shown in Figure 3e–g, the solid-state Raman spectra of all three peroxo-POMs display strong bands in the high-frequency region (ca. 930–980 cm^–1^), which are attributed to the ν­(W=O) stretching vibrations. The ν­(O–O) stretching vibrations of coordinated peroxo groups appear around 850–870 cm^–1^, in good agreement with reported peroxotungstates.^3c,10c–10f^ Moreover, the ν­(W–O_peroxo_) stretching vibrations can be observed in the 500–650 cm^–1^ region, corresponding to motions within the {W­(O_2_)} units.^17^

**Figure 3:**
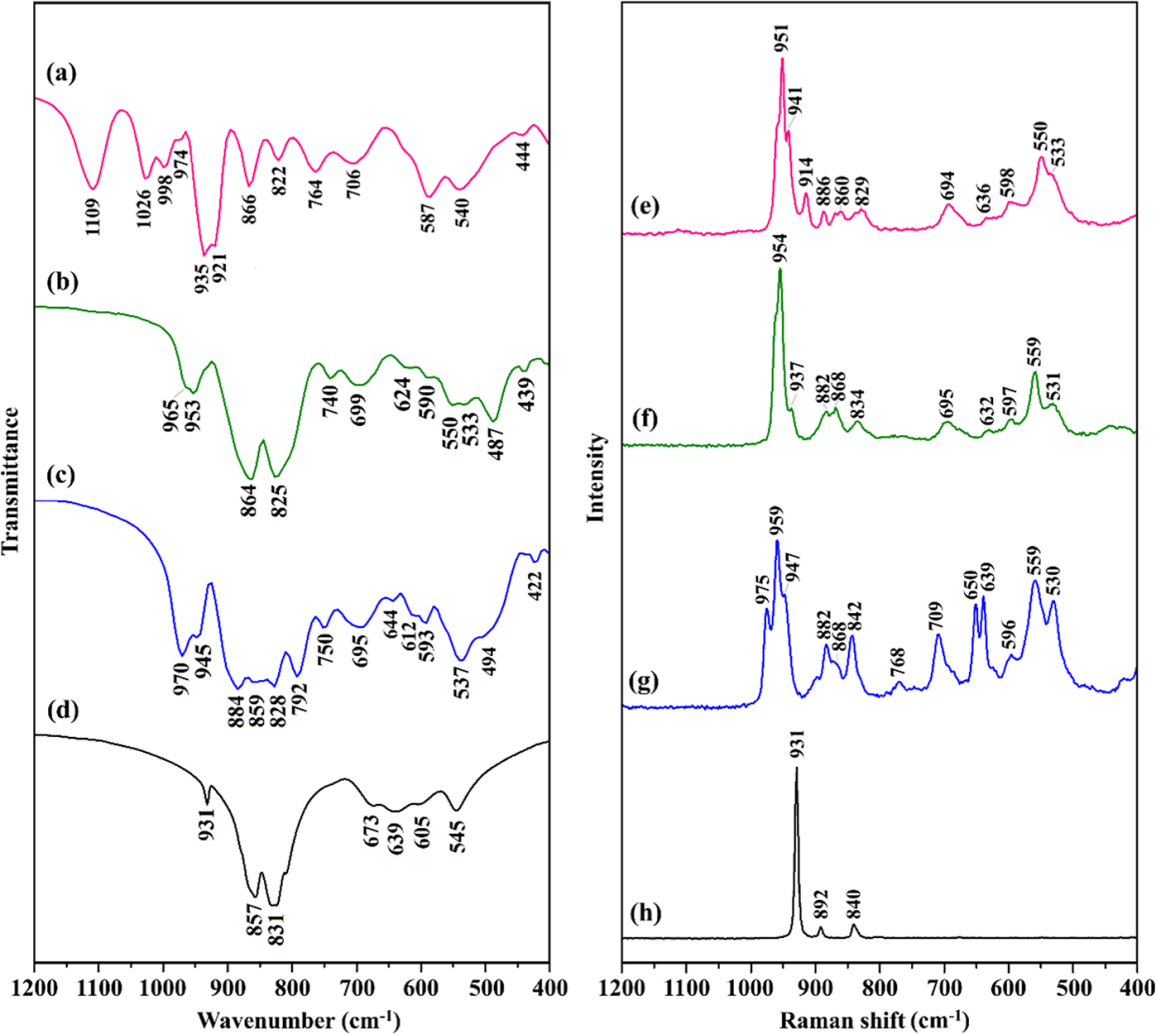
FT-IR spectra of (a) **KNa-P**_**2**_ **W**_**6**_, (b) **K-As**_**2**_ **W**_**6**_, (c) **KNa-Me**_**2**_ **As**_**2**_ **W**_**6**_, and (d) Na_2_WO_4_ precursor, and solid-state Raman spectra of (e) **KNa-P**_**2**_ **W**_**6,**_ (f) **K-As**_**2**_ **W**_**6**_, (g) **KNa-Me**_**2**_ **As**_**2**_ **W**_**6**_, and (h) Na_2_WO_4_ precursor, all recorded in the 1200–400 cm^–1^ region.

### NMR Spectroscopy

We used multinuclear NMR spectroscopy to investigate the stability behavior of the novel polyanions **P**_**2**_ **W**_**6**_, **As**_**2**_ **W**_**6**_, and **Me**_**2**_ **As**_**2**_ **W**_**6**_ in solution. The ^31^P­{^1^H} NMR spectrum of **KNa-P**_**2**_ **W**_**6**_ in H_2_O (pH 6) shows two resonances at 5.89 and 1.40 ppm, the latter corresponding to free phosphate (confirmed by H_3_PO_4_ at pH 6) arising from partial hydrolysis of the polyanion (Figure S4). With increasing measurement time, additional resonances appear, suggesting progressive decomposition of the **P**_**2**_ **W**_**6**_ species in H_2_O. Notably, only a single sharp resonance at 5.85 ppm is observed after dissolving **KNa-P**_**2**_ **W**_**6**_ in 2 M NaCl solution, demonstrating enhanced stabilization of the intact polyanion under high ionic strength (Figure 4a, bottom spectrum). We also measured ^31^P­{^1^H} NMR of the freshly prepared reaction solution (see Experimental Section). From Figure S5, the immediate formation of **P**_**2**_ **W**_**6**_ polyanion in the solution as the predominant species can be seen. The stability of **P**_**2**_ **W**_**6**_ was monitored (in 2 M NaCl) over the course of 1 week. The spectra remained mainly unchanged, confirming the long-term existence of the polyanion without significant decomposition. Furthermore, the pH-dependence studies indicated that **P**_**2**_ **W**_**6**_ remains stable at pH 5–7, but decomposition occurs upon lowering the pH to 4. Remarkably, readjustment of the solution back to pH 6 results in regeneration of the intact **P**_**2**_ **W**_**6**_ species as shown in Figure 4b.

**Figure 4:**
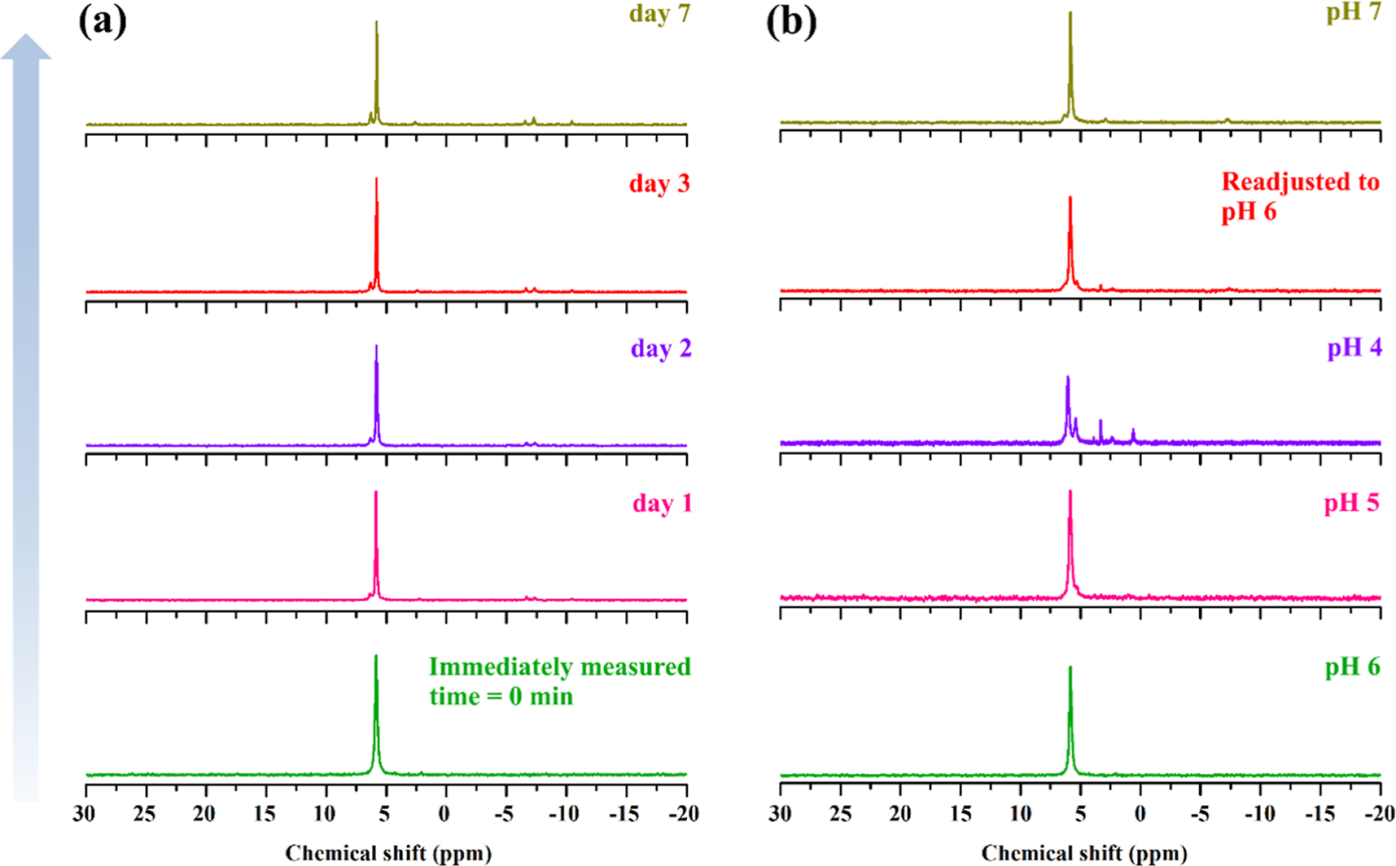
^31^P­{^1^H} NMR spectra of **KNa-P**_**2**_ **W**_**6**_ dissolved in 2 M NaCl: (a) time-dependent measurements at pH 6, recorded immediately after dissolution and after 1–7 days; (b) pH-dependent measurements upon acidification from pH 6 to 4 with 1 M HCl, followed by readjustment to pH 6 and 7 with 1 M NaOH.

The ^183^W NMR spectrum of **KNa-P**_**2**_ **W**_**6**_ in 2 M NaCl at pH 6 displays three resonances at −174, −529, and −574 ppm with an intensity ratio of 1:1:1 (Figure 5), consistent with *C*_2_ symmetry of the polyanion in the solid state. The two upfield resonances at −529 and −574 ppm correspond to the tungsten centers of the edge-sharing {WO_7_} units, each coordinated by peroxo ligands. The downfield peak at −174 ppm is attributed to the {WO_6_} tungsten atoms with terminal oxo ligands. The observed chemical shifts are consistent with the previously reported values for peroxo- and oxo-tungstate species.^1a,3c,18^ Similarly, we observed three resonances in the ^183^W NMR spectrum of **K-As**_**2**_ **W**_**6**_ (dissolved in H_2_O) at −163, −511, and −554 ppm (Figure S6).

**Figure 5:**
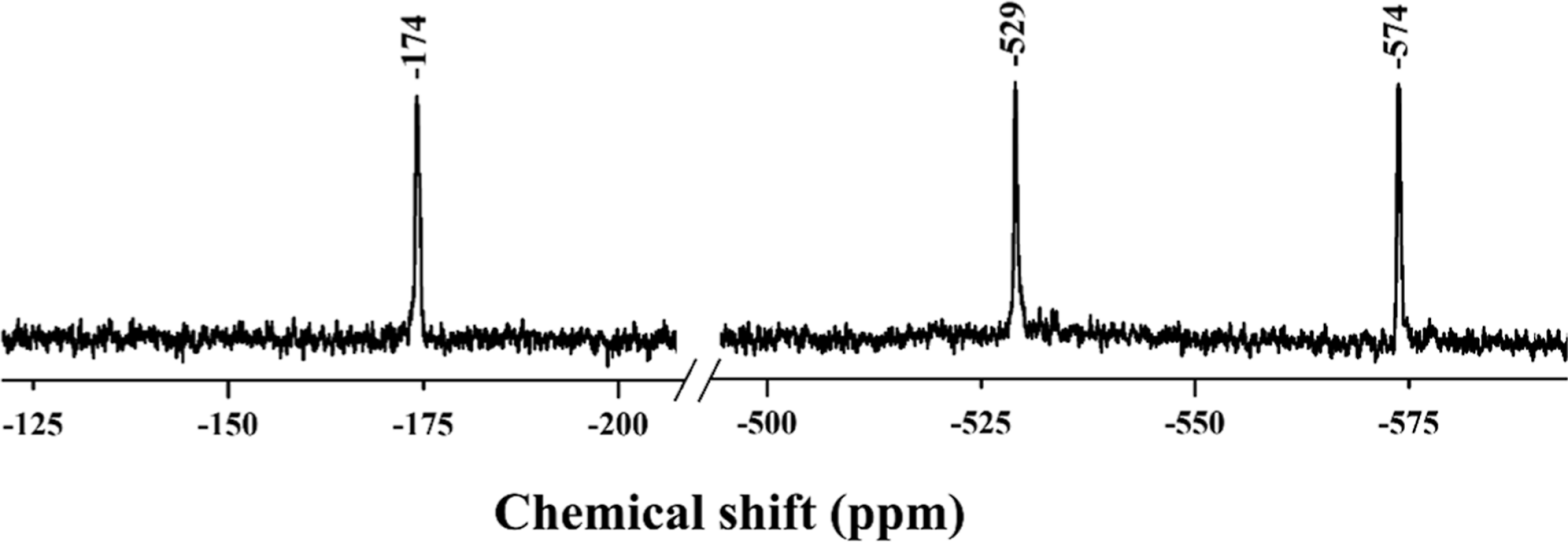
Room-temperature ^183^W NMR spectrum of **KNa-P**_**2**_ **W**_**6**_ dissolved in 2 M NaCl at pH 6.

NMR spectroscopy was further used to probe the solution behavior of the organoarsonate­(V) derivative **Me**_**2**_ **As**_**2**_ **W**_**6**_. As depicted in Figure S7, the ^1^H and ^13^C­{^1^H} NMR spectra of **Me**_**2**_ **As**_**2**_ **W**_**6**_ were recorded in H_2_O at pH 6 and displayed a single resonance at 1.4 ppm (^1^H) and 15.2 ppm (^13^C­{^1^H}), respectively, due to the two structurally equivalent methyl groups. The chemical shifts of the free methyl arsonate ligand in the same solvent at the same pH appear at 1.7 (^1^H) and 16.9 ppm (^13^C­{^1^H}), respectively (Figure S7). ^13^C­{^1^H} NMR of the freshly prepared reaction solution demonstrates that **Me**_**2**_ **As**_**2**_ **W**_**6**_ is formed as the predominant species, with the peak observed at 15.5 ppm (Figure S8). Time-dependent NMR studies show no spectral changes, indicating that the {CH_3_AsO_3_} groups remain coordinated and that **Me**_**2**_ **As**_**2**_ **W**_**6**_ retains its structural integrity in aqueous solution for at least 2 weeks (Figure S9a). pH-dependent measurements further show that **Me**_**2**_ **As**_**2**_ **W**_**6**_ is stable at pH 5–6, while decomposition occurs at pH 3–4. Notably, restoring the pH from 3 to 6 results in regeneration of the polyanion, similar to **P**_**2**_ **W**_**6**_, highlighting the reversible and pH-responsive nature of this system (Figure S9b).

### Biological Activity Screening

POMs have been extensively investigated in biomedicine as antibacterial,^19^ antiviral,^20^ and anticancer agents,^21^ as well as for antidiabetic^22^ and anti-Alzheimer applications^23^ due to their high negative charge, large size, redox properties, and stability in aqueous solution. In addition, peroxo-functionalized complexes of transition metals such as vanadium, niobium, and tungsten have been reported to exhibit antibacterial activity,^24^ inhibit HIV-1 protease,^25^ and show insulin-mimetic effects.^26^ Motivated by these examples, we examined the antibacterial properties of **K-As**_**2**_ **W**_**6**_ and **KNa-Me**_**2**_ **As**_**2**_ **W**_**6**_ against both Gram-negative and Gram-positive bacterial strains. Unlike the phosphate analogue, these two polyanions display good stability in water. The Gram-negative set included *Pseudomonas putida* (BB 10), *Escherichia coli* (DH5α), and *Pantoea agglomerans* (CC 3570), while the Gram-positive group comprised *Bacillus megaterium* (CC 3576), *Bacillus subtilis* (CC 1919), and *Bacillus aquimaris* (MB 11). All cultures were maintained in LB medium and incubated under standard aerobic growth conditions (see Experimental Section).

The results of the biological tests revealed that both **As**_**2**_ **W**_**6**_ and **Me**_**2**_ **As**_**2**_ **W**_**6**_ display antibacterial activity against *P. putida* with MIC ≤7.8 μg/mL, indicating remarkable potency even at very low concentrations. Under identical conditions, these peroxo-POMs were inactive against the other tested Gram-negative and Gram-positive strains, suggesting a strain-specific mode of action. It should be noted that these findings are based on a limited number of tested strains, and strain-specific responses may vary. Notably, the reference polyanions [*A*-α-As^V^W_9_O_34_]^9–^ and [As^III^_2_W_19_O_67_(H_2_O)]^14–^ showed no antibacterial effect. Also, selected standard antibiotics were tested as positive controls, exhibiting MIC values of 7.8 μg/mL for ampicillin, 2.8 μg/mL for chloramphenicol, and 1.4 μg/mL for both tetracycline and kanamycin (Table 2). Comparable inhibitory effects were previously reported for the tetra-organoantimony-functionalized 18-tungsto-2-arsenate­(V) [(PhSb^III^)_4_(*A*-α-As^V^W_9_O_34_)_2_]^10–^.^27^ This suggests that incorporation of lipophilic Sb­(III)-aryl groups can also enhance bacterial uptake or interactions with cell membranes. As summarized in Table 2, derivatives with fewer organometallic groups display substantially higher MIC values, indicating reduced efficacy. The decrease in activity can be rationalized by lower lipophilicity, steric hindrance, or limited accessibility of the active sites in polyanions.^27–29^ Overall, the results suggest that high antimicrobial potency can arise from two complementary strategies: (i) introduction of reactive peroxo-groups that may promote oxidative stress or alter electronic properties, and (ii) incorporation of organometallic moieties that enhance lipophilicity and facilitate interactions with bacterial cells. Both approaches appear to be effective when applied to tungstoarsenates. However, additional structure–activity relationship investigations are required to gain deeper insight into the mechanistic basis.

**Table 2:** MIC Determination of Various POM Salts and Selected Standard Antibiotics Against the Growth of *Pseudomonas putida* Bacteria

**POM-salt** **or standard antibiotic**	**MIC determination** **(μg/mL)**	**ref.**
K_6_[{(HAs^V^O_4_)W_3_O_7_(O_2_)_2_}_2_O]	<7.8^a^	this work
K_3.3_Na_2.7_[{(CH_3_As^V^O_3_)W_3_O_7_(O_2_)_2_}_2_O]	<7.8^a^	this work
Cs_6.5_Na_3.5_[(PhSb^III^)_4_(*A*-α-As^V^W_9_O_34_)_2_]	<7.8^a^	^27^
Rb_9.25_Na_0.75_[(Sb^III^OH)_4_(*A*-α-As^V^W_9_O_34_)_2_]	1000	
Cs_4.5_Na_7.5_[(PhSb^III^)_3_(*B*-α-As^III^W_9_O_33_)_2_]	15.6	^28^
Cs_4.5_K_5.5_[(PhSb^III^)_2_As_2_^III^W_19_O_67_(H_2_O)]	62.5	
Cs_3_K_3.5_Na_4.5_[(PhSb^III^){Na(H_2_O)}As^III^_2_W_19_O_67_(H_2_O)]	125	
Cs_3_KNa_6_[Na{2-(Me_2_HN^+^CH_2_)C_6_H_4_Sb^III^}As^III^_2_W_19_O_67_(H_2_O)]	500	^29^
Rb_2.5_K_5.5_[{2-(Me_2_HN^+^CH_2_)C_6_H_4_Sb^III^}_2_As^III^_2_W_19_O_67_(H_2_O)]	1000	
Na_9_[*A*-α-As^V^W_9_O_34_]	no inhibition	^27^
K_14_[As^III^_2_W_19_O_67_(H_2_O)]	no inhibition	^29^
Ampicillin^b^	7.8	this work
Chloramphenicol^b^	2.8	this work
Tetracycline^b^	1.4	this work
Kanamycin^b^	1.4	this work

a No bacterial growth was found at the minimum concentration (7.8) tested.

b Concentration of 100 μg/mL was used as a positive control.

## Conclusions

In summary, we report on the synthesis of three novel peroxo-containing heteropolyanions, **P**_**2**_ **W**_**6**_, **As**_**2**_ **W**_**6**_ and **Me**_**2**_ **As**_**2**_ **W**_**6**_, obtained in aqueous solution via the stoichiometric reaction of Na_2_WO_4_ with the corresponding heterogroup precursor in the presence of H_2_O_2_ at pH 6. Careful control of the reagent stoichiometry, ionic strength, and solution pH was essential for the formation and isolation of the pure [{XW_3_O_7_(O_2_)_2_}_2_O]^6–^ species. The three polyanions **P**_**2**_ **W**_**6**_, **As**_**2**_ **W**_**6**_ and **Me**_**2**_ **As**_**2**_ **W**_**6**_ were characterized in the solid state by single-crystal X-ray diffraction, TGA, elemental analysis, FT-IR, and Raman spectroscopy. Moreover, comprehensive multinuclear NMR spectroscopy (^1^H, ^13^C, ^31^P, and ^183^W) confirmed the structural integrity of all polyanions in solution within a pH window of 5–7. At pH values below 5, decomposition was observed; however, readjustment of the pH to 6 led to reformation of the intact polyanion, highlighting the reversible and pH-responsive nature of this system. Preliminary biological evaluations revealed that **As**_**2**_ **W**_**6**_ and **Me**_**2**_ **As**_**2**_ **W**_**6**_ display selective antibacterial activity against *P. putida*, suggesting potential for further investigation into their mode of action and structure–activity relationships.

## Supplementary Material



## Notes

Synthesis of orthoarsenic acid, FT-IR and NMR spectra, thermograms, and bond valence sum calculations (PDF)
