# Supplementary material for: Peroxo-Containing Heteropolyanions [{XW3O7(O2)2}2O]6– (X = HPO4 2–, HAsO4 2–, CH3AsO3 2–): Synthesis, Structure, and Antibacterial Properties
Source: Inorg Chem. 2026 May 19;65(21):11867–75. doi: 10.1021/acs.inorgchem.6c01165 (PMC13231414; doi:10.1021/acs.inorgchem.6c01165)
Supplement: Supplementary file 1 [file ic6c01165_si_001.pdf]

Supporting Information

Peroxo-Containing Heteropolyanions $[\{XW_3O_7(O_2)_2\}_2O]^{6-}$ ($X = HPO_4^{2-}$, $HAsO_4^{2-}$, $CH_3AsO_3^{2-}$): Synthesis, Structure, and Antibacterial Properties

Sahar Khandan,[a] Anupam Sarkar,[a] Arun Pal,[a] Friedrich Matteo Lüderitz,[a] Marziyeh Kianihaftlang,[a] Ayush Kant Ranga,[a] Levente Kiss,[b] Matthias S. Ullrich,[a] Arnulf Materny,[a] Cristian Silvestru,[b] and Ulrich Kortz*,[a]

[a] School of Science, Constructor University, Campus Ring 1, 28759 Bremen, Germany.

[b] Department of Chemistry, Supramolecular Organic and Organometallic Chemistry Centre (SOOMCC), Faculty of Chemistry and Chemical Engineering, Babeş-Bolyai University, 11 Arany Janos, 400028 Cluj-Napoca, Romania.

E-mail address: ukortz@constructor.university

Synthesis of orthoarsenic acid (H_3AsO_4)

Caution! The reaction involves the formation of nitrogen oxide gases and must therefore be performed in a well-ventilated fume hood equipped with an appropriate gas-trapping setup.

Arsenic trioxide (As_2O_3, 100 g, 0.50 mol) was combined with concentrated HNO_3 (100 mL, 1.44 mol, 65%) in a ground-joint flask under stirring. The flask outlet was connected to a gas-bubbling trap containing concentrated H_2SO_4, which effectively captured the evolved nitrogen oxides as nitrosyl hydrogen sulfate. The mixture was heated to ~130 °C for 1 h, until the evolution of nitrogen oxides ceased. After cooling to room temperature, the reaction mixture was filtered through a sintered glass funnel to remove unreacted As_2O_3. The filtrate contained H_3AsO_4, and its concentration was determined by titration against 5 M KOH, using phenolphthalein as an indicator.

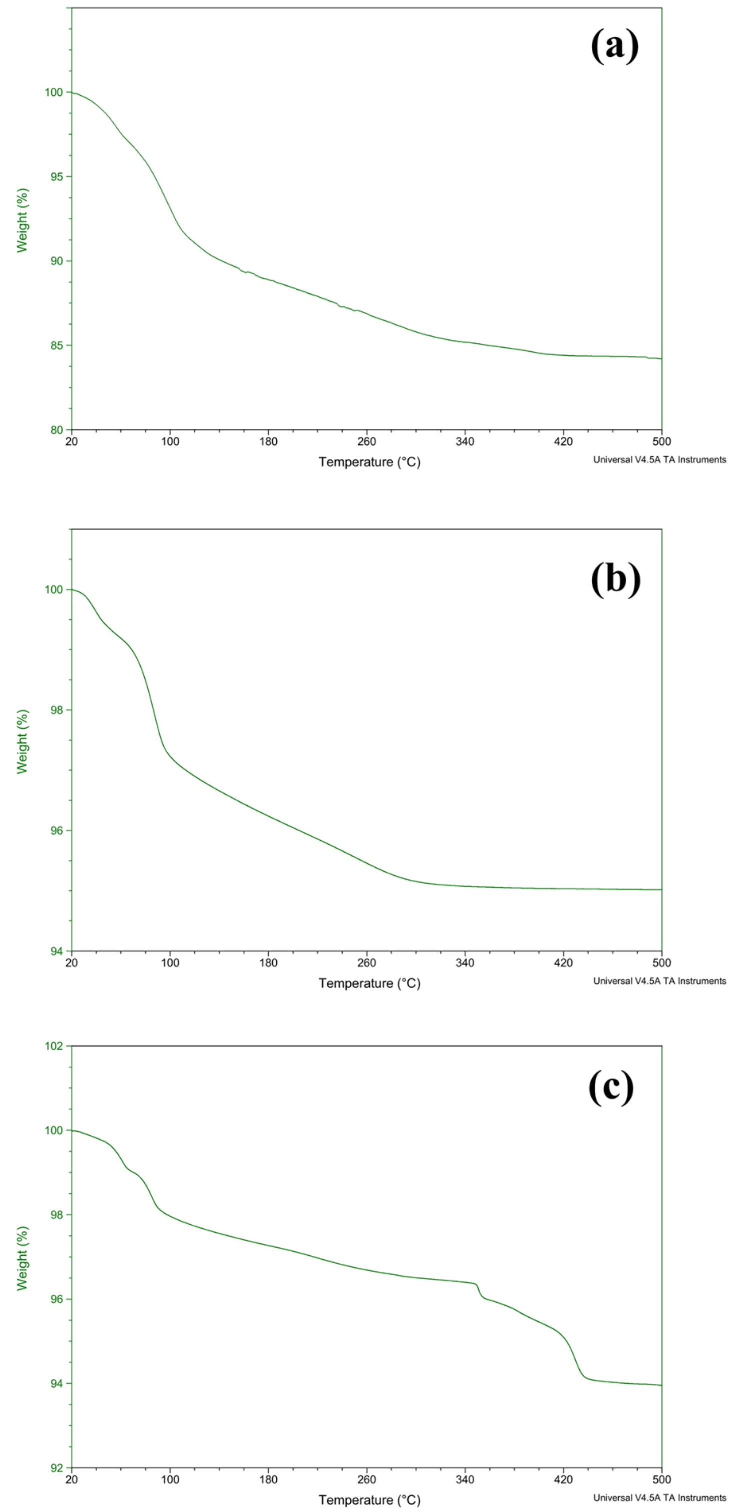

Figure S1. Thermogram of (a) **KNa-P_2W_6**, (b) **K-As_2W_6**, and (c) **KNa-$Me_2As_2W_6$** recorded from room temperature to 500 ºC, under N_2.

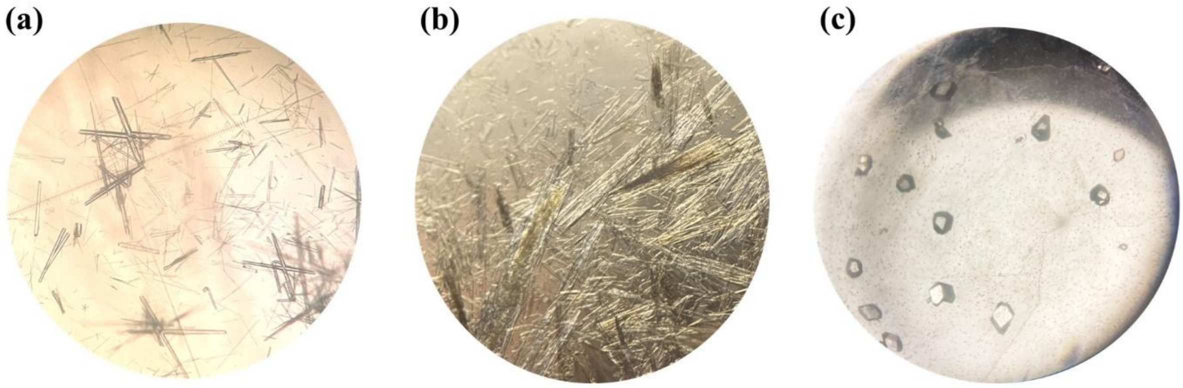

Figure S2. Crystal morphology of (a) **KNa-P_2W_6**, (b) **K-As_2W_6**, and (c) **KNa-$Me_2As_2W_6$**, observed under the microscope.

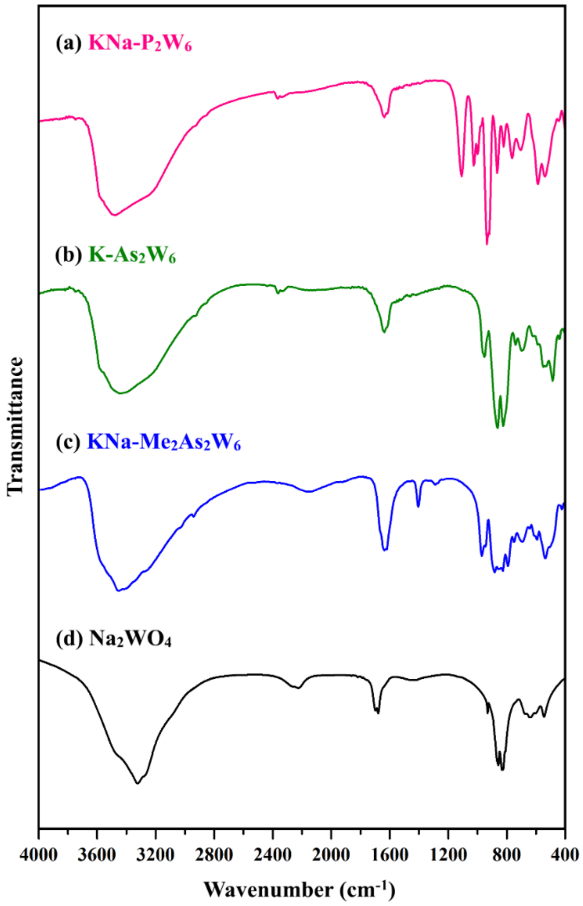

Figure S3. FT-IR spectra of (a) **KNa-P_2W_6**, (b) **K-As_2W_6**, (c) **KNa-$Me_2As_2W_6$**, and (d) Na_2WO_4 precursor, recorded in the range of 4000-400 cm^{-1}.

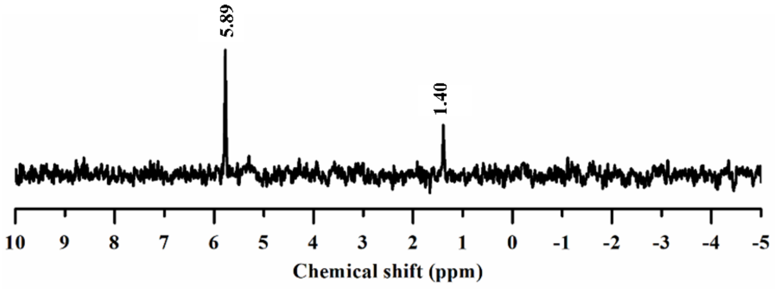

Figure S4. $^{31}P\{^{1}H\}$ NMR spectrum of **KNa-P_2W_6** dissolved in water at pH 6.

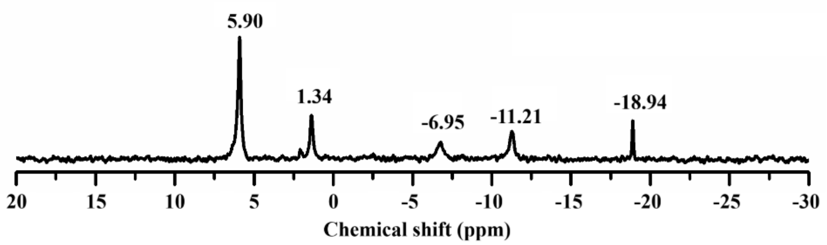

Figure S5. $^{31}P\{^{1}H\}$ NMR spectrum of the freshly prepared reaction solution of **KNa-P_2W_6** at pH 6.

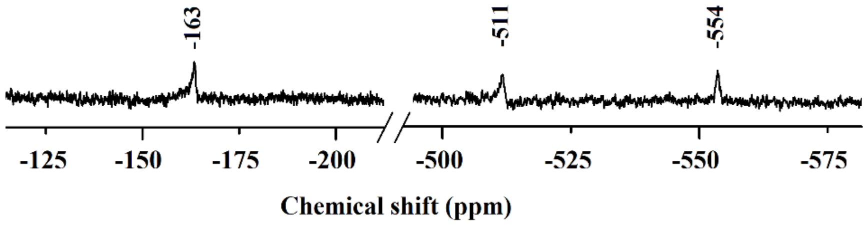

Figure S6. Room temperature ^{183}W NMR spectrum of **K-As_2W_6** dissolved in water at pH 6.

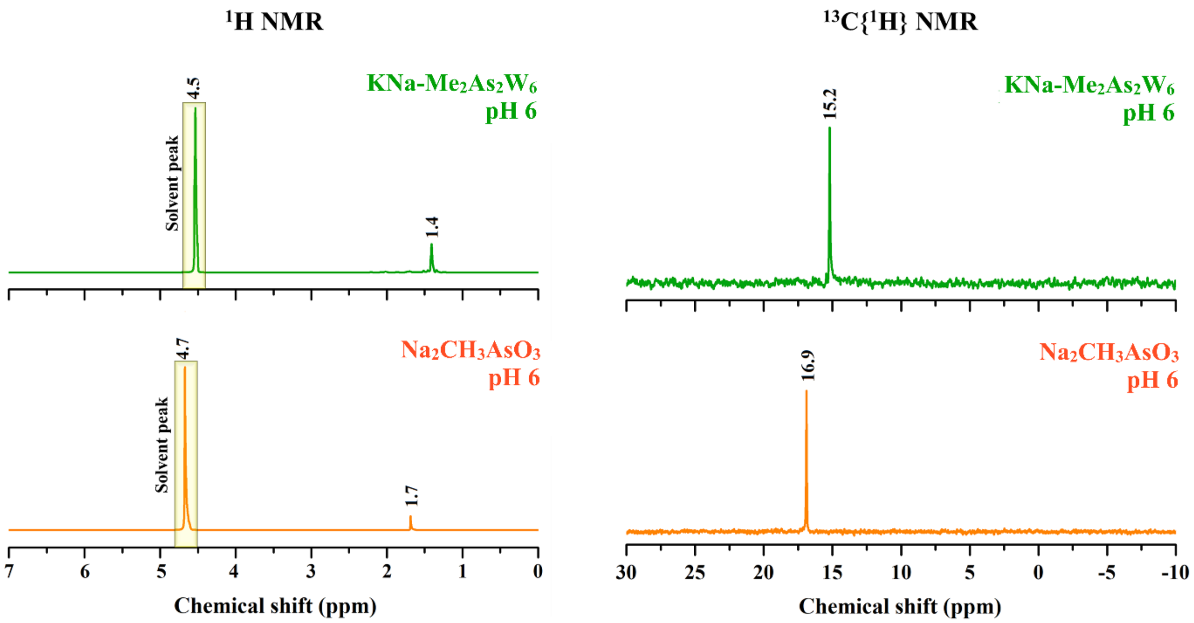

Figure S7. ^{1}H and $^{13}C\{^{1}H\}$ NMR spectra of **KNa-Me$_2$As$_2$W$_6$** and $Na_2CH_3AsO_3$ precursor dissolved in water at pH 6.

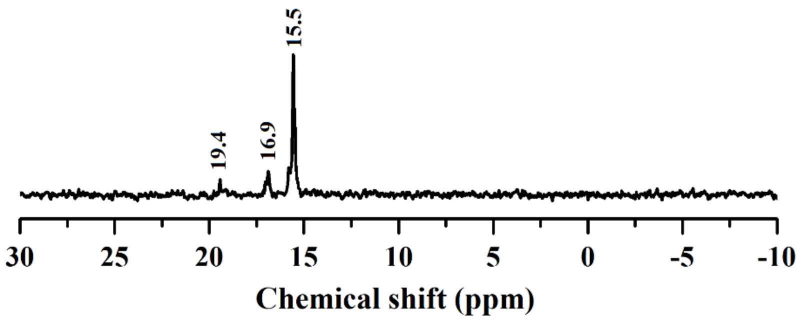

Figure S8. $^{13}C\{^{1}H\}$ NMR spectrum of the freshly prepared reaction solution of **KNa-Me$_2$As$_2$W$_6$** at pH 6.

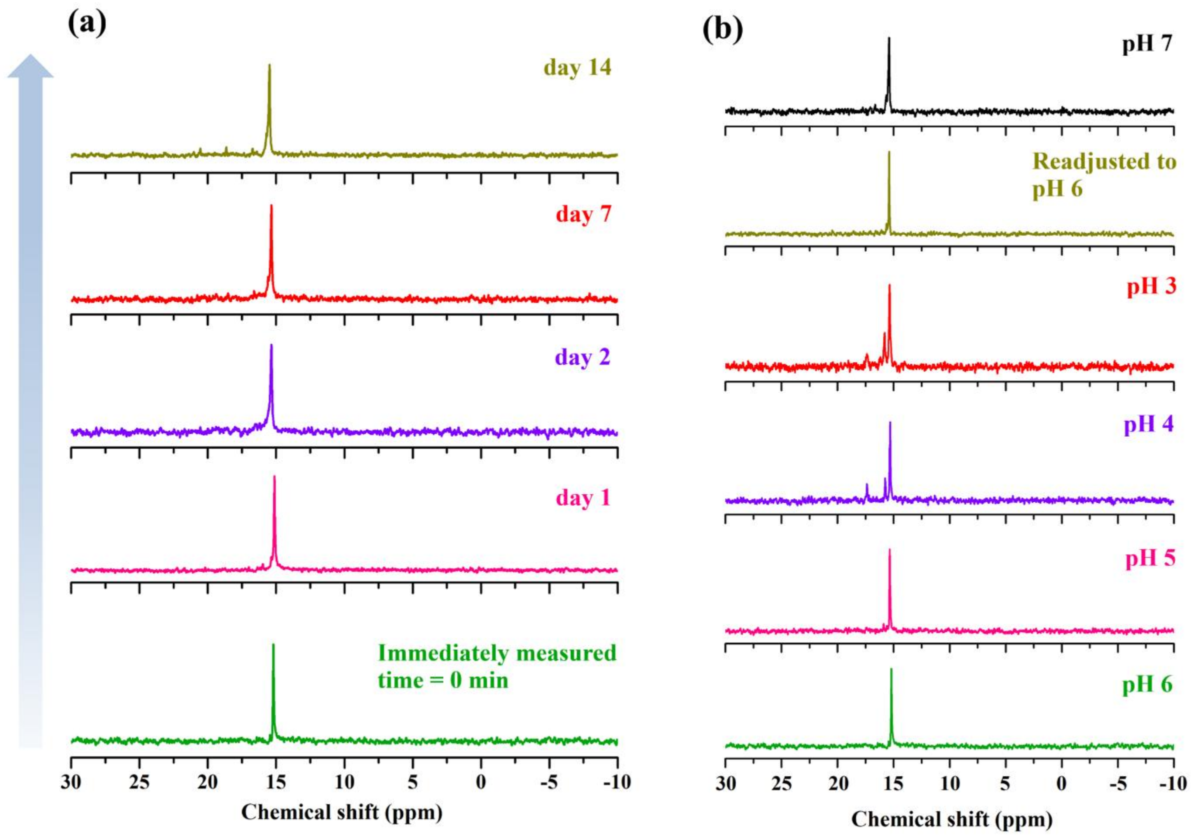

Figure S9. $^{13}C\{^{1}H\}$ NMR spectra of **KNa-Me$_2$As$_2$W$_6$** dissolved in water: (a) time-dependent measurements at pH 6, recorded immediately after dissolution and after 1-14 days; (b) pH-dependent measurements upon acidification from pH 6 to 4 with 1 M HCl, followed by re-adjustment to pH 6 and 7 with 1 M NaOH.

Table S1. Bond valence sum* values for phosphorus atoms, and for the bridging and terminal oxygen atoms in the structure of polyanion $[\{(HP^{V}O_4)W_3O_7(O_2)_2\}_2O]^{6-}$.

Atom	Bond distance (Å)	BVS value	Atom type
P1	P1—O1P1 (1.572)<br>P1—O2P1 (1.529)<br>P1—O3P1 (1.555)<br>P1—O4P1 (1.509)	4.919	P(V)
P2	P2—O1P2 (1.546)<br>P2—O2P2 (1.553)<br>P2—O3P2 (1.537)<br>P2—O4P2 (1.544)	4.860	P(V)
O12	W1—O12 (2.028)<br>W2—O12 (1.832)	2.021	Oxo
O15	W1—O15 (1.967)<br>W5—O15 (1.966)	1.769	Oxo
O23	W2—O23 (1.922)<br>W3—O23 (2.017)	1.769	Oxo
O26	W2—O26 (1.901)<br>W6—O26 (1.931)	2.029	Oxo
O34	W3—O34 (1.942)<br>W4—O34 (1.966)	1.830	Oxo
O46	W4—O46 (2.032)<br>W6—O46 (1.823)	2.044	Oxo
O56	W5—O56 (2.202)<br>W6—O56 (1.855)	1.663	Oxo
O156	W1—O156 (2.009)<br>W5—O156 (2.048)<br>W6—O156 (2.153)	2.032	Oxo
O234	W2—O234 (2.148)<br>W3—O234 (2.023)<br>W4—O234 (1.997)	2.115	Oxo
O4P1	P1—O4P1 (1.509)	1.339	Hydroxo
O4P2	P2—O4P2 (1.544)	1.218	Hydroxo
O1T	W1—O1T (1.728)	1.685	Oxo
O2T	W2—O2T (1.727)	1.689	Oxo
O3T	W3—O3T (1.734)	1.658	Oxo
O4T	W4—O4T (1.725)	1.698	Oxo
O5T	W5—O5T (1.713)	1.754	Oxo
O6T	W6—O6T (1.721)	1.717	Oxo

* $BVS = \sum_{\mathrm{i}} \exp\left(\frac{R_0 - R_i}{b}\right)$, where R_0 is the empirical bond valence parameter specific to the bond type, R_i is the measured bond length for bond, and b is the empirical constant, typically 0.37 Å.

Table S2. Bond valence sum* values for arsenic atoms, and for the bridging and terminal oxygen atoms in the structure of polyanion $[\{(HAs^{V}O_4)W_3O_7(O_2)_2\}_2O]^{6-}$.

Atom	Bond distance (Å)	BVS value	Atom type
As1	As1—O1A1 (1.687)<br>As1—O2A1 (1.684)<br>As1—O3A1 (1.699)<br>As1—O4A1 (1.656)	5.044	As(V)
As2	As2—O1A2 (1.715)<br>As2—O2A2 (1.667)<br>As2—O3A2 (1.697)<br>As2—O4A2 (1.651)	5.037	As(V)
O12	W1—O12 (1.889)<br>W2—O12 (2.011)	1.874	Oxo
O14	W1—O14 (1.937)<br>W4—O14 (1.912)	1.982	Oxo
O16	W1—O16 (1.833)<br>W6—O16 (2.026)	2.021	Oxo
O23	W2—O23 (1.954)<br>W3—O23 (1.952)	1.834	Oxo
O34	W3—O34 (2.044)<br>W4—O34 (1.813)	2.056	Oxo
O45	W4—O45 (1.919)<br>W5—O45 (2.018)	1.775	Oxo
O56	W5—O56 (1.945)<br>W6—O56 (1.992)	1.763	Oxo
O123	W1—O123 (2.178)<br>W2—O123 (2.026)<br>W3—O123 (1.986)	2.091	Oxo
O456	W4—O456 (2.159)<br>W5—O456 (2.030)<br>W6—O456 (1.991)	2.098	Oxo
O4As1	As1—O4As1 (1.656)	1.349	Hydroxo
O4As2	As2—O4As2 (1.651)	1.368	Hydroxo
O1T	W1—O1T (1.726)	1.693	Oxo
O2T	W2—O2T (1.715)	1.745	Oxo
O3T	W3—O3T (1.728)	1.684	Oxo
O4T	W4—O4T (1.727)	1.689	Oxo
O5T	W5—O5T (1.719)	1.726	Oxo
O6T	W6—O6T (1.712)	1.759	Oxo

* $BVS = \sum_{i} \exp\left(\frac{R_0 - R_i}{b}\right)$, where R_0 is the empirical bond valence parameter specific to the bond type, R_i is the measured bond length for bond, and b is the empirical constant, typically 0.37 Å.

Table S3. Bond valence sum* values for arsenic atoms, and for the bridging and terminal oxygen atoms in the structure of polyanion $[\{(CH_3As^VO_3)W_3O_7(O_2)_2\}_2O]^{6-}$.

Atom	Bond distance (Å)	BVS value	Atom type
As1	As1—C1 (1.880)<br>As1— O1A1 (1.708)<br>As1— O2A1 (1.685)<br>As1— O3A1 (1.686)	5.012	As(V)
As2	As2—C2 (1.899)<br>As2— O1A2 (1.717)<br>As2— O2A2 (1.682)<br>As2— O3A2(1.687)	4.923	As(V)
O1A1	W1— O1A1 (2.102)<br>As1— O1A1 (1.708)	1.786	Oxo
O2A1	W2— O2A1 (2.238)<br>As1— O2A1 (1.685)	1.672	Oxo
O3A1	W3— O3A1 (2.197)<br>As1— O3A1 (1.686)	1.719	Oxo
O1A2	W4— O1A2 (2.092)<br>As2— O1A2 (1.717)	1.774	Oxo
O2A2	W5— O2A2 (2.223)<br>As2— O2A2 (1.682)	1.700	Oxo
O3A2	W6— O3A2 (2.218)<br>As2— O3A2 (1.687)	1.689	Oxo
O12	W1— O12 (1.907)<br>W2— O12 (1.986)	1.877	Oxo
O14	W1— O14 (1.906)<br>W4— O14 (1.902)	2.094	Oxo
O16	W1— O16 (1.810)<br>W6— O16 (2.046)	2.063	Oxo
O23	W2— O23 (1.973)<br>W3— O23 (1.993)	1.692	Oxo
O34	W3— O34 (2.041)<br>W4— O34 (1.806)	2.087	Oxo
O56	W5— O56 (1.961)<br>W6— O56 (1.991)	1.725	Oxo
O123	W1— O123 (2.137)<br>W2— O123 (2.043)<br>W3— O123 (1.988)	2.111	Oxo
O456	W4— O456 (2.154)<br>W5— O456 (2.045)<br>W6— O456 (1.994)	2.068	Oxo

* $BVS = \sum_i \exp\left(\frac{R_0 - R_i}{b}\right)$, where R_0 is the empirical bond valence parameter specific to the bond type, R_i is the measured bond length for bond, and b is the empirical constant, typically 0.37 Å.

Table S4. Average W–O_{peroxo}, O–O, and X[a]–O bond lengths in **P_2W_6**, **As_2W_6**, and **$Me_2As_2W_6$**.

Polyanion	Average bond distance (Å)		
	X[a]–O	O–O	W–O_{peroxo}
**P_2W_6**	1.54(3)	1.48(6)	1.96(6)
**As_2W_6**	1.68(2)	1.49(3)	1.96(3)
**$Me_2As_2W_6$**	1.69(4)	1.48(4)	1.96(8)

[a] X denotes P in **P_2W_6** and As in **As_2W_6** and **$Me_2As_2W_6$**.
